# Diagnostic Delay in Desquamative Gingivitis: An Observational Cohort Study

**DOI:** 10.1111/odi.70276

**Published:** 2026-03-19

**Authors:** Alessandra Caggiula, Francesca Romano, Elena Dentico, Fabiana Pinto, Maria Pia Lopez‐Jornet, Massimo Petruzzi

**Affiliations:** ^1^ School of Dentistry University of Bari “Aldo Moro” Bari Italy; ^2^ Instituto Murciano de Investigación Biosanitaria (IMIB‐Arrixaca‐UMU) Clínica Odontológica Universitaria Hospital Morales Meseguer Murcia Spain

**Keywords:** desquamative gingivitis, diagnostic delay, non‐plaque induced gingivitis, oral lichen planus, pemphigoid, pemphigus vulgaris

## Abstract

**Objectives:**

Non‐plaque‐induced gingivitis encompasses a heterogeneous group of conditions as defined in the 2017 Classification of Periodontal and Peri‐Implant Diseases and Conditions. Recognition is challenging and often leads to diagnostic delay. This study aimed to evaluate diagnostic delay in a cohort of patients with non‐plaque‐induced gingivitis presenting as desquamative gingivitis, and to identify associated factors.

**Materials and Methods:**

In this observational study, patients were recruited from public and private oral medicine centres. Participants completed a questionnaire collecting demographic, social, and clinical information related to their condition. Association between variables and diagnostic delay were analyzed using the Student's *t*‐test (significance *p* < 0.05).

**Results:**

Eighty‐six patients (43 from private and 43 from public centres) were enrolled. The mean diagnostic delay was 10 months. No statistically significant associations were observed between diagnostic delay and age, gender, symptom presence, smoking habits, or definitive diagnosis. In contrast, diagnostic delay was significantly higher in private oral health centre patients (11.19 months vs. 8.75 months *p* < 0.05) and in patients living more than 34 km from the diagnostic centre (*p* < 0.05).

**Conclusions:**

Proximity to specialized diagnostic centres and the type of centre (public vs. private) were identified as determinants to reduce diagnostic delay in patients with desquamative gingivitis.

## 1 Introduction

Non–plaque‐induced gingivitis (NPIGs) is an umbrella term encompassing a wide range of pathological conditions affecting the gingival mucous membranes, which share similar clinical manifestations. NPIGs often present as desquamative gingivitis (DG), a condition clinically characterized by diffuse erythema, ulcerations, erosions, vesicles, or desquamation of the marginal and attached gingiva (Shaqman et al. 2020). It primarily affects the vestibular gingiva, particularly its marginal portion, although in some cases the entire thickness of the attached gingiva may be involved. Vesicles, blisters, erosions, ulcers, pseudomembranes, and erythema may occur individually or in combination, and can sometimes extend to the alveolar mucosa (Lo Russo et al. 2008). Extraoral mucocutaneous sites, including the larynx, conjunctiva, esophagus, nasal and genital mucosa, and skin, may be affected either synchronously or metachronously. DG can also represent a prodromal sign or an early manifestation of a systemic disease (Sciuca et al. 2022).

Cases of NPIGs presenting as DG require a thorough and specialized diagnostic workup to assess a broad differential diagnosis, including autoimmune‐mediated diseases such as lichen planus, mucous membrane pemphigoid, pemphigus vulgaris, erythema multiforme, lupus erythematosus, chronic ulcerative gingivitis, and lichenoid reactions. Allergic and pseudo‐allergic reactions must also be considered, including plasma cell gingivitis, contact allergic reactions, and oral allergy syndrome (Holmstrup et al. 2018; Mortazavi et al. 2024).

Diagnosing DG is often challenging due to the difficulty in differentiating between pathological entities with nearly indistinguishable clinical features (Daltaban et al. 2020). Therefore, immunological tests (e.g., ELISA, indirect immunofluorescence), microbiological assessments (e.g., cultures, genetic analysis), and tissue examinations (e.g., histopathology, direct immunofluorescence, immunohistochemistry) are frequently required (Petruzzi et al. 2021, 2022). Final diagnosis is typically established after an extensive clinical and laboratory process, often resulting in a more or less significant diagnostic delay.

Patients with DG are generally referred to specialized oral pathology and oral medicine centres, mostly located within public healthcare institutions (e.g., universities, hospitals, research centres), with limited access in private practice settings. A notable gap exists in private dental practices concerning the recognition and management of oral mucosal lesions. Evidence suggests that 85% of private dentists report difficulties in diagnosing such conditions (Ergun et al. 2009). Furthermore, 93% neither perform biopsies nor consult colleagues when uncertain, and 62% do not regularly review current scientific literature to update their knowledge (Ergun et al. 2009). Supporting this concern, a study by Kondori et al. (2011) reported that 43% of oral lesion diagnoses made by referring dentists were incorrect.

López‐Jornet et al. (2008) compared the diagnostic performance of dentists with 5 years of clinical experience to that of dentists without professional experience, demonstrating higher diagnostic sensitivity and specificity among the more experienced practitioners. Similarly, a recent Italian study by Sbricoli et al. (2024) assessed dentists' ability to identify oral mucosal lesions according to their level of clinical experience. Overall diagnostic accuracy was approximately 50%. Specifically, correct identification of malignant lesions ranged from 52% to 60%, paradoxically lower among more experienced dentists, whereas accuracy for benign lesions ranged from 55% to 71% and was higher among experienced practitioners (Sbricoli et al. 2024). Clinical challenges in managing oral mucosal lesions have also been reported by Friesen et al. (2019) and Farah et al. (2008), who observed that in approximately 75% of cases, with an average of two medical consultations per patient. Despite multiple specialist consultations, only 28.5% of patients receive a provisional diagnosis that is subsequently confirmed by an oral medicine specialist (Coppola et al. 2021). Furthermore, Coppola et al. (2021) reported that a substantial proportion of patients referred to Oral Medicine Units present with immune‐mediated diseases, accounting for approximately 40% of cases.

Petruzzi et al. (2021) reviewed the most commonly misdiagnosed autoimmune oral lesions, reporting a mean diagnostic delay of 312 days for reticular OLP and 452 days for erosive atrophic OLP, and approximately 7 months for pemphigus and pemphigoid.

Diagnostic inaccuracy in these pathologies is particularly critical in DG, where delayed diagnosis can significantly impact prognosis. Misdiagnosis or delayed diagnosis may lead to inappropriate pharmacological, surgical, or dental interventions, exposing patients to unnecessary treatments, potential side effects, and worsening of their condition. Identifying and addressing the factors associated with diagnostic delays is therefore essential for improving clinical outcomes, informing healthcare policy in dentistry, and enhancing patients' quality of life (Hassona et al. 2019).

Patients with DG frequently report a history of repeated and closely spaced oral hygiene sessions, including scaling and root planing, without significant improvement of the inflammatory process (Mignogna et al. 2001). Periodontal specialist support is certainly beneficial in achieving therapeutic goals by applying appropriate techniques and technologies at the right time (Scattarella et al. 2011). Mistreatments and diagnostic delays negatively impact both quality of life and disease progression (Mignogna et al. 2001; Ergun et al. 2009).

Hassona et al. reported a mean delay of 5.5 months in their cross‐sectional study on oral autoimmune blistering diseases. They emphasized the concept that “diagnosis of DG in often challenging because of the no specific features in the large number of conditions that present as gingival inflammation, the most common being plaque related gingivitis and Oral Lichen Planus” (Hassona et al. 2018).

The aim of the present study was to evaluate and describe the diagnostic delay in a cohort of patients affected by NPIGs presenting as DG, and to analyze the factors associated with this delay.

## 2 Materials and Methods

### 2.1 Patient Recruitment and Ethics

This study was conducted in accordance with the Declaration of Helsinki of the World Medical Association (2004) and was approved by the Independent Ethics Committee of the “Azienda Ospedaliero‐Universitaria Consorziale Policlinico di Bari” (authorization number 7270).

The study adhered to the STROBE guidelines for observational studies (Supporting Information).

This observational study was conducted between November 2022 and November 2024 in both private and public oral medicine centres in the metropolitan area of Bari, Southern Italy. Eighty‐six consecutive patients with a clinical diagnosis of DG were enrolled from referrals to a public oral health centre (PuOHC) and a private oral health centre (PrOHC). The same specialist in Oral Pathology and Medicine (M.P.) diagnosed all patients from both PuOHC and PrOHC. After the definitive diagnosis, patient's data were collected retrospectively. The two centres operate independently.

### 2.2 Inclusion and Exclusion Criteria

Inclusion criteria were as follows: (i) patients diagnosed with NPIGs due to Oral Lichen Planus according to the American Academy of Oral Medicine (Cheng et al. 2016), Pemphigus Vulgaris, or Pemphigoid (diagnosis confirmed by pathological exam and direct immunofluorescence) presenting as DG (Petruzzi et al. 2021); (ii) patients age ≥ 18 years or older; (iii) patients who provided informed consent to participate in the study; (iv) patients compliant with the proposed protocol.

Exclusion criteria were as follows: (i) patients with plaque‐induced gingivitis; (ii) patients who refused to participate in the study; (iii) non‐cooperative patients; (iv) patients younger than 18 years; (v) pregnant patients; (vi) patients with necrotizing ulcerative gingivitis.

### 2.3 Anamnestic Questionnaire

The anamnestic questionnaire collected demographic data (sex, age, and place of residence) as well as lifestyle habits, such as alcohol consumption (yes/no) and tobacco use (yes/former/no). Duration of smoking or alcohol consumption was not recorded, nor was the time since cessation for former smokers. Symptoms were recorded for each patient and categorized as a dichotomous variable (yes/no).

### 2.4 Diagnostic Delay

To quantify the diagnostic delay, patients were asked to report, in months, the time gap between the onset of symptoms and/or signs and the first visit to the centre that subsequently provided the correct final diagnosis. The assessment of diagnostic delay was therefore calculated from the day following the 15th, according to the Italian guidelines in dentistry by the Health Ministry (https://www.salute.gov.it/).

### 2.5 Calculation of Distance Between Patient's Residence and Diagnostic Centre

The calculation of the distance between patients' residences and the diagnostic centre (PuOHC and PrOHC) that established the final diagnosis was made using Google Maps (https://www.google.com/maps). The patient's residential address and the address of the centre where the final diagnosis was made were entered. The shortest driving route in kilometers was considered.

### 2.6 Statistical Analysis

Diagnostic delay across the entire sample was evaluated in relation to demographic variables (age, sex), place of residence, behavioral factors (smoking and alcohol consumption), and clinical characteristics (presence or absence of symptoms). Normality of continuous variables was assessed using the Shapiro–Wilk test and graphical inspection. Given the sample size and the robustness of parametric tests to moderate deviations from normality, Student's *t*‐test was applied with a significance level of *p* < 0.05 (StataCorp18—StataCorp LLC).

**FIGURE 1:**
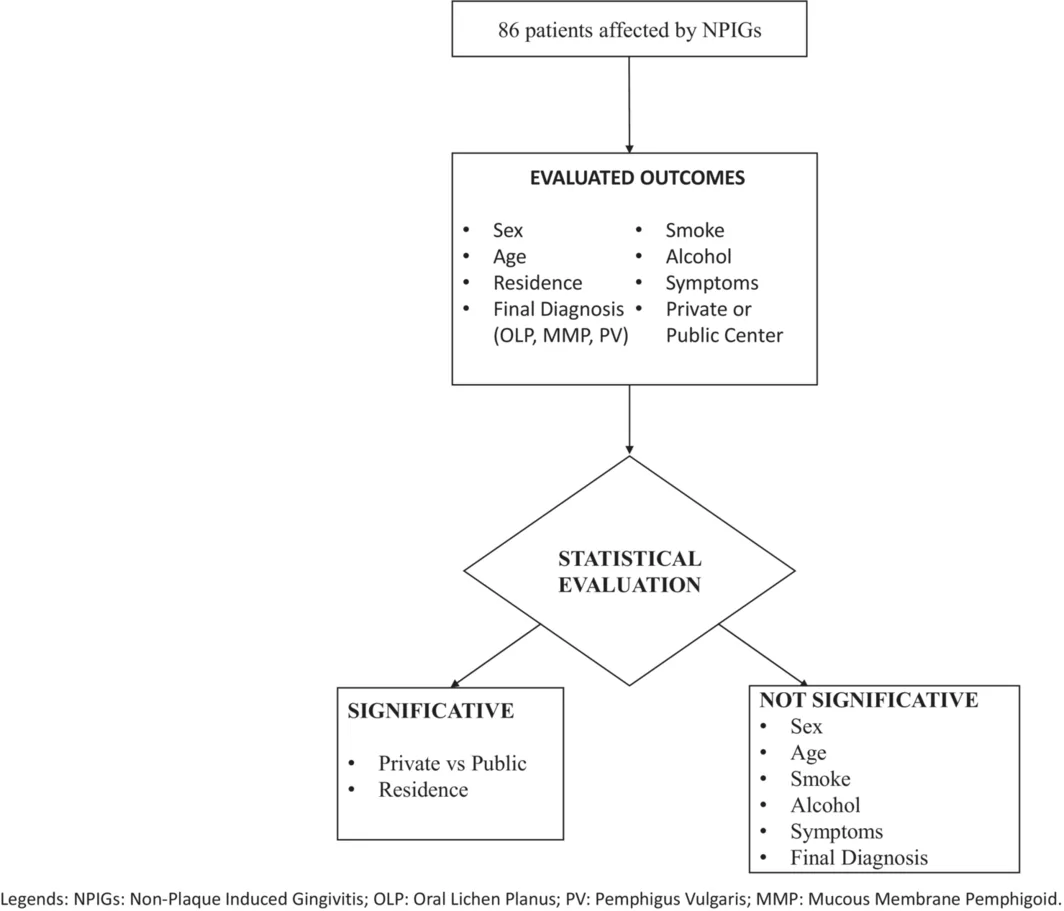
Study flowchart.

The study flowchart is shown in Figure 1.

## 3 Results

A total of 86 patients diagnosed with DG were enrolled, including 43 from PuOHC (age from 22 to 87 years, mean age 65.9 ± 13.96) and 43 from PrOHC (age from 30 to 85 years, mean age 60.13 ± 12.66). The mean age was 62.66 ± 13.77 years, with a female‐to‐male ratio of 3.3:1. Sixty‐six patients were female aging from 22 to 87 years (mean age: 62.48 ± 13.56 years), and 20 were male aging from 41 to 82 years (mean age: 63.25 ± 13.9 years).

The distribution of DG final diagnoses was: Mucous Membrane Pemphigoid in 40 patients (46.51%), Oral Lichen Planus in 38 patients (44.18%), and Pemphigus Vulgaris in 8 patients (9.30%). The overall diagnostic delay was 10.14 ± 9.72 months. The mean diagnostic delay for female patients was 10.38 ± 10.49 months, and 9.35 ± 6.91 months for male patients. This difference was not statistically significant. Patients older than 63 years experienced a mean delay of 11.71 ± 11.19 months, whereas those aged 63 years or younger had a delay of 8.61 ± 7.91 months, showing no statistically significant differences (*p* < 0.05).

Among the 25 (29%) current or former smokers, the mean diagnostic delay was 8.81 ± 7.98 months, compared to 10.7 ± 10.39 months in the 61 never‐smokers (71%). This difference was not statistically significant (*p* = 0.75). In the PuOHC the former smoker was 7 (16.28%) with a diagnostic delay of 6.39 ± 5.52 months, 22 never‐smoker with a diagnostic delay 5.62 ± 6.94 months and 14 (32.55%) smokers with a diagnostic delay of 10.07 ± 9.05 months. In the PrOHC there was 1 (2.32%) former smoker patients with a diagnostic delay of 9 months, 39 (90.7%) never‐smoker patients with a diagnostic delay of 11.61 ± 10.70 months and 3 (6.98%) current smokers with a mean diagnostic delay of 8 ± 1.73 months.

For non‐alcohol users, the mean diagnostic delay was 10.91 ± 10.90 months, compared to 8.88 ± 7.42 months in alcohol users. This difference was without statistical significance. In the PuOHC the no‐alcohol users were 23 (53.48%) with an average diagnostic delay of 7.58 ± 7.94 months, the alcohol users were 20 (46.52%) with an average diagnostic delay of 6.56 ± 7.69 months. In the PrOHC the no‐alcohol users were 28 (66.1%) with an average diagnostic delay of 12.42 ± 11.92 months, the alcohol users were 15 (34.9%) with an average diagnostic delay of 9.2 ± 5.74 months.

Three (3.49%) patients were former smokers and alcohol‐users with an average diagnostic delay of 4 ± 2.64 months and 9 (20.93%) current smokers and alcohol‐users with an average diagnostic delay of 10.25 ± 9.75 months.

No relationship was found between the final diagnosis and consumption habits.

Sixty‐one patients (71%) reported one or more symptoms, with a diagnostic delay of 9.79 ± 8.19 months. In contrast, the 25 asymptomatic patients (29%) experienced a longer diagnostic delay of 10.86 ± 12.5 months. However, this difference was not statistically significant.

The average distance from the patient's residence to the diagnostic centre was 34.15 ± 32.41 km. Patients living ≤ 34 km away had a mean delay of 7.93 ± 8.77 months, while those living > 34 km away experienced a delay of 11.95 ± 10.19 months. This difference was statistically significant (*p* < 0.05).

The diagnostic delay for patients referred to the PuOHC was 8.75 ± 14.93 months, compared to 11.21 ± 10.35 months for those seen at the PrOHC. This difference was statistically significant (Supporting information).

**FIGURE 2:**
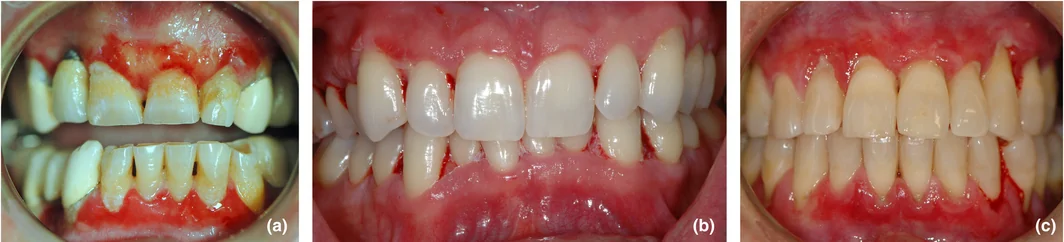
Cases of desquamative gingivitis in (a) Mucous Membrane Pemphigoid, (b) Oral Pemphigus Vulgaris, (c) Oral Lichen Planus.

The final diagnosis did not significantly influence the diagnostic delay: Oral lichen planus: 9.75 ± 11.01 months; Mucous Membrane Pemphigoid: 10.71 ± 9.21 months; Pemphigus Vulgaris: 8 ± 6.44 months. In Figure 2 are shown three cases of DG relative to Mucous Membrane Pemphigoid, Pemphigus Vulgaris, and Oral Lichen Planus.

In the PuOHC 16 (37.21%) patients received the final diagnosis of Oral Lichen Planus with an average diagnostic delay of 6.6 ± 8.02 months; the diagnosis of Mucous Membrane Pemphigoid was made in 21 (48.84%) patients with an average age of 7.65 ± 8.35 months; 6 (13.95%) patients were diagnosed as Pemphigus Vulgaris with an average diagnostic delay of 6.54 ± 5.51 months.

**FIGURE 3:**
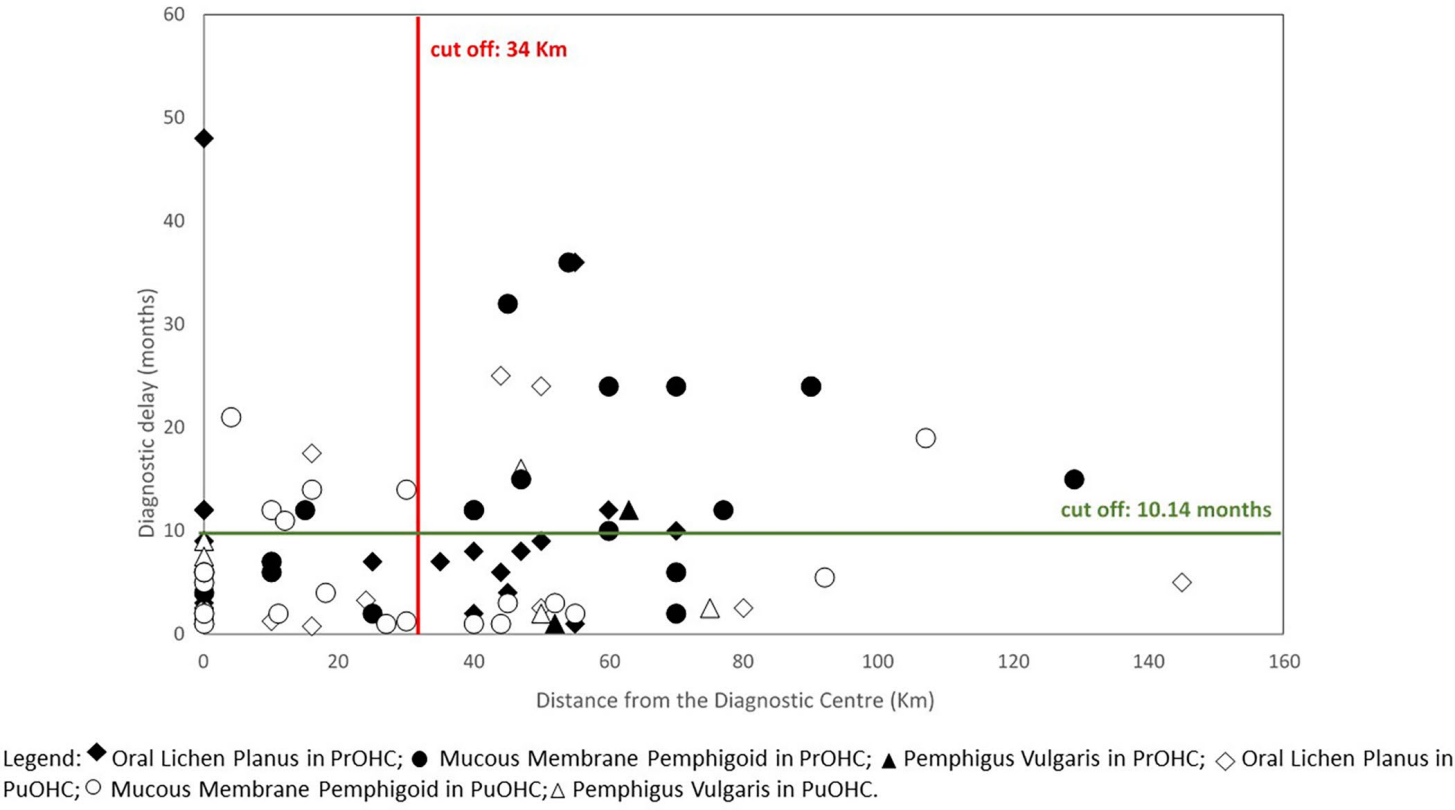
Scattergram representing the distribution of patients affected by desquamative gingivitis in relation to diagnostic delay and distance from the diagnostic center.

In the PrOHC the final diagnosis of Oral Lichen Planus was made in 22 (51.16%) with a diagnostic delay of 9.54 ± 9.25 months; 19 (44.19%) patients received the diagnosis of Mucous Membrane Pemphigoid with a diagnostic delay of 13.31 ± 9.24 months; the diagnosis of Pemphigus Vulgaris was made in 2 (4.65%) patients with a diagnostic delay of 6.5 ± 7.77 months. Table 1 summarized the aforementioned results while Figure 3 illustrates the associations between diagnostic delay and distance from the diagnostic centre, type of diagnostic centre, and DG final diagnosis.

**TABLE 1:** Variables associated with desquamative gingivitis diagnostic delay.

	Sex		Age (years)		Smoke		Alcohol		Symptoms		Distance (km)		Pathologies			Center	
	M	F	< 63	> 63	Yes—Former	No	Yes	No	Yes	No	< 34	> 34	MMP	OLP	PV	PuOHC	PrOHC
N° Patients (86)	20	66	43	43	25	61	35	51	61	25	43	43	40	38	8	43	43
DD (mths ± ds)	9.35 ± 6.9	10.38 ± 10.49	8.61 ± 7.91	11.71 ± 11.19	8.81 ± 7.98	10.7 ± 10.39	8.88 ± 7.42	10.91 ± 10.91	9.79 ± 8.19	10.86 ± 12.50	7.93 ± 8.77	11.95 ± 10.19	10.71 ± 9.21	9.75 ± 11.01	8 ± 6.44	8.75 ± 14.93	11.19 ± 10.35
	*p* = 0.95		*p* = 0.15		*p* = 0.75		*p* = 2.49		*p* = 0.48		***p* < 0.00001**		*p* > 0.05			***p* = 0.047**	

*Note:* In bold significative *p* values.

Abbreviations: DD, Diagnostic Delay; MMP, Mucous Membrane Pemphigoid; OLP, Oral Lichen Planus; PrOHC, Private Oral Health Center; PuOHC, Public Oral Health Center; PV, Pemphigus Vulgaris.

## 4 Discussion

This study revealed that NPIGs presenting as DG were diagnosed with an average delay of approximately 10 months. This timeframe is questionable, given the diagnostic complexity of defining diagnosis, limited access to oral specialists, and the commonly needed biopsy.

Whether such delay is acceptable should be evaluated from a medico‐legal perspective, considering both the impact on patient quality of life and the efficiency of the healthcare system (Albano et al. 2024). While the diagnostic timeline for Oral Squamous Cell Carcinoma is well‐established, no similar temporal guidelines exist for oral autoimmune disorders (Marella et al. 2018; Petruzzi et al. 2023).

Although females were more frequently affected, the factors associated with diagnostic delays included the distance between the patient's residence and the diagnostic oral health centre, as well as the type of centre (private or public) where the final diagnosis was made.

PuOHC may exhibit shorter diagnostic delays than private practices, possibly due to greater accessibility, collaboration with university centres, and the presence of a substantial staff dedicated almost exclusively to this service. Furthermore, public centres are generally easier to reach, being well connected to transportation infrastructure. Evaluating diagnostic delay in these patients may help develop interceptive strategies and facilitate earlier referral to appropriate facilities for managing DG, as reported in previous studies on diagnostic delays in autoimmune oral diseases (Petruzzi et al. 2023).

Previous studies correlating smoking and alcohol consumption with diagnostic delay have primarily focused on potentially malignant and neoplastic lesions of the oral cavity. Specifically, no definitive data exist linking alcohol or tobacco use to diagnostic delays, particularly in oral autoimmune diseases (Brouha et al. 2005; Swaminathan et al. 2024). It could be hypothesized that non‐smokers and non‐drinkers are more attentive to health‐related issues, leading them to notice early signs and symptoms in the oral mucosa. However, this hypothesis requires confirmation through appropriately designed studies.

While the concept of diagnostic delay originates from oncology, it is equally relevant to chronic medical conditions such as Crohn's disease, rheumatoid arthritis, and Sjögren's syndrome. It is well established that early diagnosis of chronic diseases improves clinical outcomes, slows disease progression, and significantly enhances patients' quality of life (Al‐Karadsheh et al. 2024).

Developing effective chairside strategies for the early identification of DG is essential: one recent example is the modified Nikolsky's sign, a simple and rapid test to detect vesiculobullous conditions affecting the oral mucosa (Petruzzi 2024). Additionally, serological testing to identify circulating autoantibodies—common in many DG—has become a reliable and routine part of diagnostic workups (Vaillant et al. 2000).

The issue of diagnostic delay in DG remains underreported in the literature. Shaqman et al. investigated diagnostic delays in 41 patients with gingival symptoms, reporting a mean delay of 10.8 months before a definitive diagnosis—consistent with our findings (Shaqman et al. 2020). In contrast, Vaillant et al. (2000) reported a longer mean delay of 19 months in a retrospective study of 33 patients with DG.

To the best of our knowledge, this is the largest study to date on the topic, with the highest number of patients analyzed and the first to assess differences in diagnostic delay between public and private healthcare services. We also highlighted the critical role of geographical accessibility: patients living further from diagnostic centres were more likely to experience delayed diagnoses. As previously reported, the use of telemedicine and digital platforms could reduce consultation times and improve access for patients in rural or underserved areas (Petruzzi and De Benedittis 2016).

The main limitations of this study are those inherent to observational research. Socioeconomic status, a known determinant of healthcare access, could not be assessed due to patients' reluctance to disclose income data. In fact, a lower socioeconomic status may enable patients to afford specialist visits at private centres and cover travel expenses to reach the site where the diagnosis is made. Furthermore, the phenomenon of “diagnostic wandering”—in which patients consult multiple healthcare providers before reaching a specialized centre—has not been evaluated in detail (Hassona et al. 2018; Raha et al. 2023). Future studies could investigate whether the phenomenon of diagnostic wandering is attributable to a true “patient delay” or rather to a “system delay,” caused by dentists delaying referrals to specialized centres, or to a combination of both factors.

Moreover, several factors may directly influence diagnostic delay, including the availability of oral medicine specialists, the profile of the first clinician to evaluate the lesions, the necessity for a biopsy, and, importantly, patient recall bias. This latter aspect, also referred to as “recall bias,” was partially mitigated by reviewing prior medical prescriptions presented by patients; however, the use of structured questionnaires, as demonstrated by Schmier et al. and Barsky, could further minimize this bias (Schmier and Halpern 2004; Barsky 2002). Despite these limitations, our findings are partially generalizable and provide a valid reference point for future investigations.

This study has implications for clinical practice, education, and research. Clinically, there is a clear need for timely referral of patients with DG who do not respond to conventional periodontal therapy.

The establishment of PuOHC (hospitals, university clinics, public outpatient clinics) widely distributed across the territory would facilitate patient consultation, reducing the risk of diagnostic delays. Public and private investments, along with awareness from health policy makers, could contribute to this public health initiative (Jo et al. 2020; World Health Organization 2010).

Educationally, graduate and postgraduate training programs for dentists, physicians, and dental hygienists should allocate more teaching time to DG. Although less common than plaque‐induced gingivitis, NPIGs are often underdiagnosed or misdiagnosed (McCann et al. 2005).

Future research should aim to develop simple, sensitive, and specific tools for differentiating DG from plaque‐induced gingivitis at early stages (Al‐Abeedi et al. 2015; Lo Russo et al. 2009). Periodontology and oral medicine—originally a subspecialty of periodontology—should work synergistically to improve the health of the periodontium and overall patient outcomes (Askitopoulou et al. 2017). Additional studies are warranted to better understand the causes of diagnostic delay in periodontal diseases and to support the establishment of new outpatient facilities through both public and private funding.

## 5 Conclusions

NPIGs comprises a group of pathological conditions affecting the gingival mucosa that, in severe cases, can lead to a significant impairment in quality of life.

The factors most strongly influencing diagnostic delay include the distance between the patient's residence and the diagnostic centre, and the type of centre (public or private) where the diagnosis is made.

Further prospective, controlled, multicentric studies are needed to evaluate the factors involved in the diagnostic delay in DG and autoimmune disease in order to overcome the limitations identified in the present study.

## Author Contributions

**Alessandra Caggiula:** writing – original draft, investigation, funding acquisition, conceptualization, writing – review and editing, visualization, methodology, validation, formal analysis, project administration, supervision, resources, data curation, software. **Francesca Romano:** formal analysis, data curation, methodology, resources. **Elena Dentico:** formal analysis, data curation, methodology, resources. **Fabiana Pinto:** methodology, formal analysis, data curation, resources. **Maria Pia Lopez‐Jornet:** writing – review and editing, resources, data curation, supervision. **Massimo Petruzzi:** conceptualization, writing – original draft, funding acquisition, investigation, validation, methodology, visualization, writing – review and editing, project administration, software, formal analysis, data curation, supervision, resources.

## Conflicts of Interest

The authors declare no conflicts of interest.

## Supporting information


Supporting Information Figures

## Data Availability

The data that support the findings of this study are available from the corresponding author upon reasonable request.
